# Alpha-1 antitrypsin deficiency impairs lung antibacterial immunity in mice

**DOI:** 10.1172/jci.insight.140816

**Published:** 2021-02-08

**Authors:** Lena Ostermann, Regina Maus, Jennifer Stolper, Lisanne Schütte, Konstantina Katsarou, Srinu Tumpara, Andreas Pich, Christian Mueller, Sabina Janciauskiene, Tobias Welte, Ulrich A. Maus

**Affiliations:** 1 Division of Experimental Pneumology,; 2 Clinic for Pneumology, and; 3 Institute of Toxicology and Core Facility Proteomics, Hannover Medical School, Hannover, Germany.; 4 Horae Gene Therapy Center, University of Massachusetts Medical School, Worcester, Massachusetts, USA.; 5 German Center for Lung Research, partner site BREATH, Hannover, Germany.

**Keywords:** Pulmonology, Bacterial infections, Neutrophils, Proteases

## Abstract

Alpha-1 antitrypsin (AAT) is a major inhibitor of serine proteases in mammals. Therefore, its deficiency leads to protease–antiprotease imbalance and a risk for developing lung emphysema. Although therapy with human plasma-purified AAT attenuates AAT deficiency–related emphysema, its impact on lung antibacterial immunity is poorly defined. Here, we examined the effect of AAT therapy on lung protective immunity in AAT-deficient (KO) mice challenged with *Streptococcus pneumoniae*. AAT-KO mice were highly susceptible to *S. pneumoniae*, as determined by severe lobar pneumonia and early mortality. Mechanistically, we found that neutrophil-derived elastase (NE) degraded the opsonophagocytically important collectins, surfactant protein A (SP-A) and D (SP-D), which was accompanied by significantly impaired lung bacterial clearance in *S. pneumoniae*–infected AAT-KO mice. Treatment of *S. pneumoniae*–infected AAT-KO mice with human AAT protected SP-A and SP-D from NE-mediated degradation and corrected the pulmonary pathology observed in these mice. Likewise, treatment with Sivelestat, a specific inhibitor of NE, also protected collectins from degradation and significantly decreased bacterial loads in *S. pneumoniae*–infected AAT-KO mice. Our findings show that NE is responsible for the degradation of lung SP-A and SP-D in AAT-KO mice affecting lung protective immunity in AAT deficiency.

## Graphical abstract

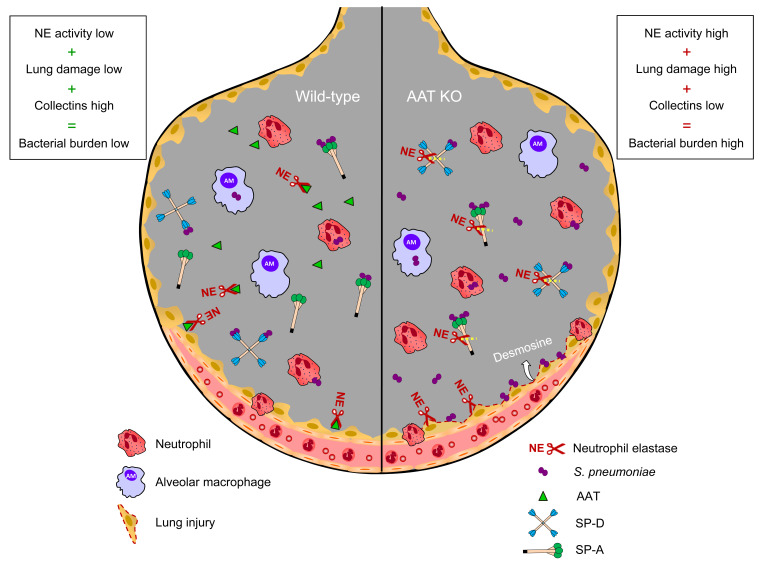

## Introduction

Alpha-1 antitrypsin (AAT) belongs to the superfamily of serine protease inhibitors (SERPINs) and is an important regulator of neutrophil elastase (NE) and cathepsin G (1, 2). Humans and mice have high serum levels of AAT, ranging at 1–2 mg/mL in humans and 3–4 mg/mL in mice, which increase upon injury or infection, thus underlining the (patho-) physiological need for sustained antiproteolytic activity in both species (1, 3, 4).

AAT is encoded by the *SERPINA1* gene and mainly produced by hepatocytes but also by monocytes/macrophages and lung epithelial cells (5–7). Over 150 mutations have been identified in the *SERPINA1* gene, some of which affect the concentration and/or functionality of AAT protein. The S (Glu264Val) and Z (Glu342Lys) mutations are most common and clinically significant and are both known to cause AAT deficiency (AATD) (2, 5, 8–11). Individuals homozygous for the Z allele exhibit roughly 90% reduced levels of circulating AAT and have an increased risk of developing chronic obstructive pulmonary disease (COPD) with emphysema (12–15). Many patients with AATD are lifelong treated with AAT augmentation therapy (16). There is clinical evidence that therapy with weekly infusions of AAT decreases emphysema progression (17, 18).

Current recommendations for AAT augmentation therapy are not based on randomized clinical trials but rather are oriented at serum AAT levels. Therefore, the standard augmentation therapy recommended for patients with AATD is defined as 60 mg AAT/kg body weight (b.w.) per week reaching approximately 50% of serum AAT levels found in healthy individuals (19). Many patients with AATD under standard augmentation therapy still experience emphysema progression. A recent prospective study revealed that double-dose augmentation therapy of 120 mg AAT/kg b.w. per week increased AAT levels to normal and reduced serine protease activities and hence markers of elastin degradation in bronchoalveolar lavage (BAL) fluids of patients with AATD (19). Results from such double-dose augmentation therapies suggest that current single-dose augmentation therapies may underestimate the need for AAT bioavailability in patients with AATD, thus calling for individualized AAT dosing considerations (19).

Since AAT deficiency is a susceptibility factor for lung damage during bacterial exacerbations (20–23), it is equally important to understand how lung protective immunity against bacterial challenge is influenced by the levels of AAT. Therefore, we examined the effect of AAT deficiency on lung protective immunity against *Streptococcus pneumoniae* in mice in the absence or presence of augmentation therapy.

## Results

### Endogenous AAT levels decrease in serum and increase in lungs in response to S. pneumoniae infection of mice.

**Figure 1:**
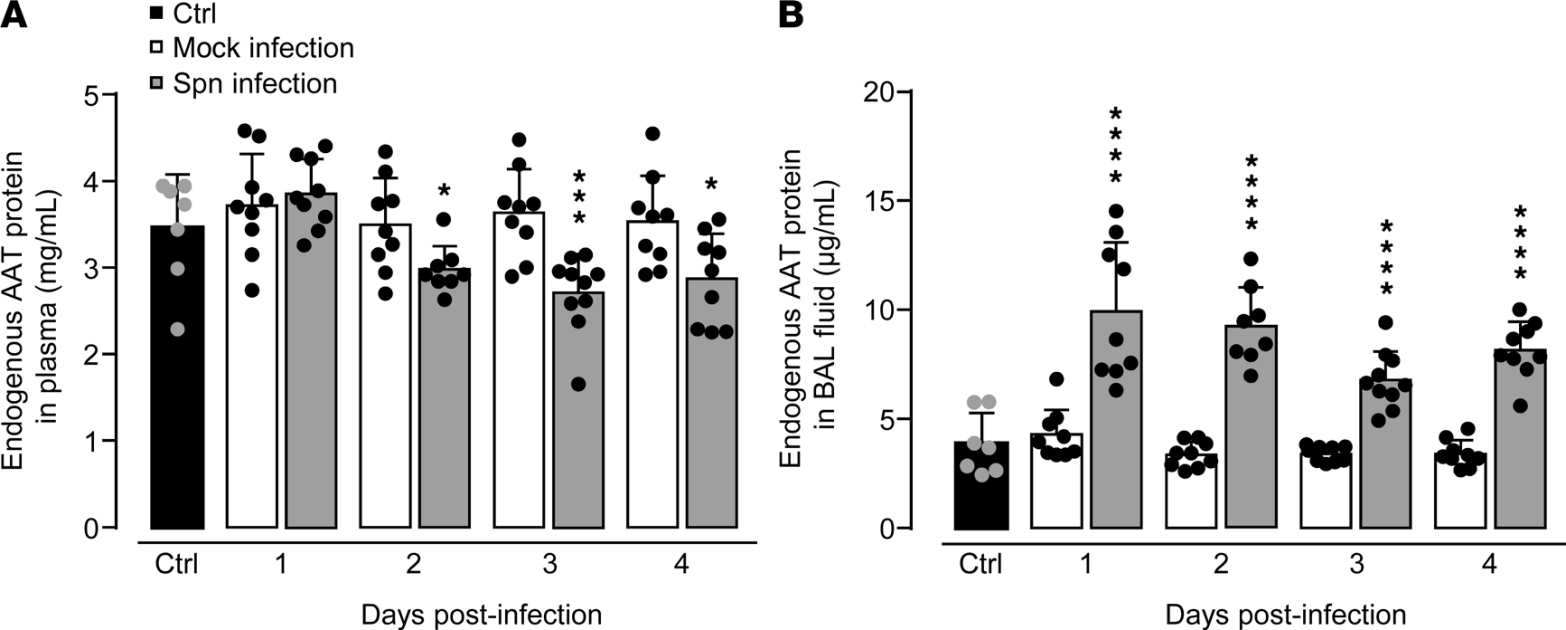
AAT protein levels in plasma and BAL fluids of WT mice infected with *S. pneumoniae.* WT mice were left untreated (black bars), were mock-infected with PBS (white bars), or were infected with *S*. *pneumoniae* (gray bars). Endogenous AAT protein was measured in plasma (**A**) and BAL fluids (**B**) of mice at days 1, 2, 3, and 4 postinfection by ELISA. Data are shown as mean ± SD of *n* = 7–9 mice per time point and treatment group and are representative of 2 independently performed experiments. *P ≤ 0.05, ***P ≤ 0.001, ****P ≤ 0.0001 compared with mock-infected mice (Mann-Whitney *U* test). Spn, *S*. *pneumoniae*.

In initial experiments, we examined the effect of pneumococcal challenge on endogenous AAT levels in WT mice. Under baseline conditions, mice showed AAT serum levels of 3–4 mg/mL, consistent with previous reports (Figure 1A) (3, 4, 24). Relative to mock infection, *S. pneumoniae*–challenged mice demonstrated approximately 25% decreased serum AAT levels on days 2–4 postinfection. At the same time, we observed significantly increased AAT levels in BAL fluid (BALF) of WT mice challenged with *S. pneumoniae* on days 1–4 postinfection (Figure 1B).

### Effect of AATD on lung antibacterial immunity.

The results from the WT mice showed that lung challenge with *S*. *pneumoniae* triggered a decrease in systemic AAT levels while pulmonary AAT levels increased in response to infection. We next examined whether AAT interferes with pneumococcal growth in vitro. However, growth of *S. pneumoniae* was not affected by 1–2.5 mg/mL of human AAT (hAAT) within 4 hours (Supplemental Figure 1; supplemental material available online with this article; https://doi.org/10.1172/jci.insight.140816DS1). Even 5 mg of hAAT/mL did not affect pneumococcal growth in vitro compared to controls (data not shown).

**Figure 2:**
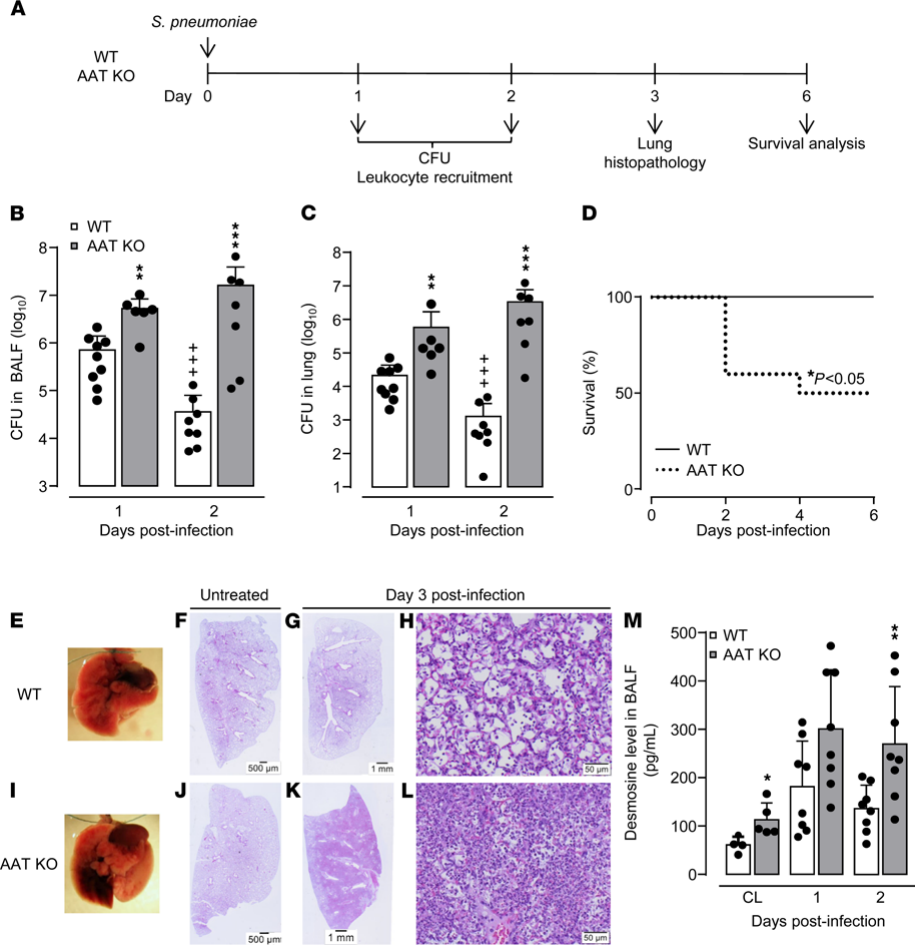
Effect of AAT deficiency on outcome in pneumococcal pneumonia. WT mice (white bars) and AAT-KO mice (gray bars) were infected orotracheally with *S*. *pneumoniae* and were analyzed at the indicated time points, as shown (**A**). (**B** and **C**) Bacterial loads in BALF (**B**) and lung tissue (**C**) at 24 hours and 48 hours postinfection. Values are shown as mean ± SD (*n* = 6–9 mice per time point and treatment group) and are representative of 2 independent experiments. (**D**) Survival analysis of *S*. *pneumoniae*–infected WT and AAT-KO mice (*n* = 9 mice per group). (**E** and **I**) Photographic illustration of lungs from *S*. *pneumoniae*–infected WT and AAT-KO mice at day 3 postinfection. (**F**–**L**) Lung histopathology of untreated (**F** and **J**, scale bars: 500 μm), or *S*. *pneumoniae*–infected WT (**G**, scale bar: 1 mm; **H** scale bar: 50 μm) or AAT-KO mice (**K**, scale bar: 1 mm; **L**, scale bar: 50 μm). Illustrations in **E**–**L** are representative of *n* = 4 mice per group. (**M**) Desmosine levels in BALF of untreated (CL) or *S*. *pneumoniae*–infected WT or AAT-KO mice, as indicated. *P ≤ 0.05, **P ≤ 0.01, ***P ≤ 0.001 compared with untreated or *S*. *pneumoniae*–infected WT mice, ^+++^*P* ≤ 0.001 compared with day 2 (Mann-Whitney *U* test, log-rank test).

Next, to reflect the clinical situation of inherited AATD, we employed recently generated AAT-KO mice lacking all 5 mouse serpin A1 paralogues, thus leading to complete AAT protein absence. AAT-KO mice were used at the age of 8–14 weeks while spontaneously developing lung emphysema only at advanced age of more than 35 weeks (25). As shown in Figure 2, AAT-KO mice responded to *S. pneumoniae* infection with significantly increased bacterial loads in BALF and lung tissue (Figure 2, B and C). Consistent with these findings, survival of AAT-KO mice was significantly decreased (by 50%) when compared with *S. pneumoniae*–infected WT mice (Figure 2D). Microscopic inspection of lungs revealed lobar pneumonia in *S. pneumoniae*–infected AAT-KO mice but not in WT mice (Figure 2, E and I). H&E-stained lung tissue sections of untreated WT and AAT-KO mice revealed a normal lung architecture with unaffected bronchi and bronchioles in both experimental groups (Figure 2, F and J); however, only the *S. pneumoniae*–infected AAT-KO mice showed severe lobar pneumonia characterized by massive interstitial and alveolar infiltration of neutrophils together with interstitial and alveolar edema and alveolar hemorrhage (Figure 2, G and H, K and L). As a marker of lung injury, we determined desmosine contents in BAL fluids of WT and AAT-KO mice at baseline and on days 1 and 2 postinfection. Desmosine is an amino acid of elastin and therefore reflects a biomarker for elastin degradation (26). Under baseline conditions (CL in Figure 2M), we found significantly higher desmosine levels in BALF of AAT-KO mice compared with WT mice. We also found substantially increased desmosine levels in BALF of *S. pneumoniae*–infected AAT-KO mice as compared with WT mice on day 2 postinfection. In parallel, AAT-KO mice revealed significantly increased neutrophil counts in BALF and lung tissue on day 3 postinfection (Supplemental Figure 2). No significant differences in numbers of alveolar macrophages and exudate macrophages were observed in WT versus AAT-KO mice after *S. pneumoniae* infection (data not shown).

### Effect of AAT augmentation therapy on lung antibacterial immunity in AAT-KO mice.

Based on the finding that *S. pneumoniae* infection of AAT-KO mice led to substantially increased lung bacterial loads along with significantly decreased survival, we next aimed to reverse the phenotype in the KO mice by systemic treatment of mice with hAAT. As shown in Supplemental Figure 3, hAAT was detectable by Western blot analysis in plasma and BALF of AAT-KO mice receiving AAT therapy at 48–72 hours postinfection (lanes 5–8) but not in AAT-KO mice receiving PBS as vehicle (lanes 1–4). Human AAT protein levels increased on days 2 and 3 postinfection both in plasma and in BALF of *S. pneumoniae*–infected AAT-KO mice receiving AAT therapy with a slight decline of AAT levels by day 3 (Supplemental Figure 3F).

**Figure 3:**
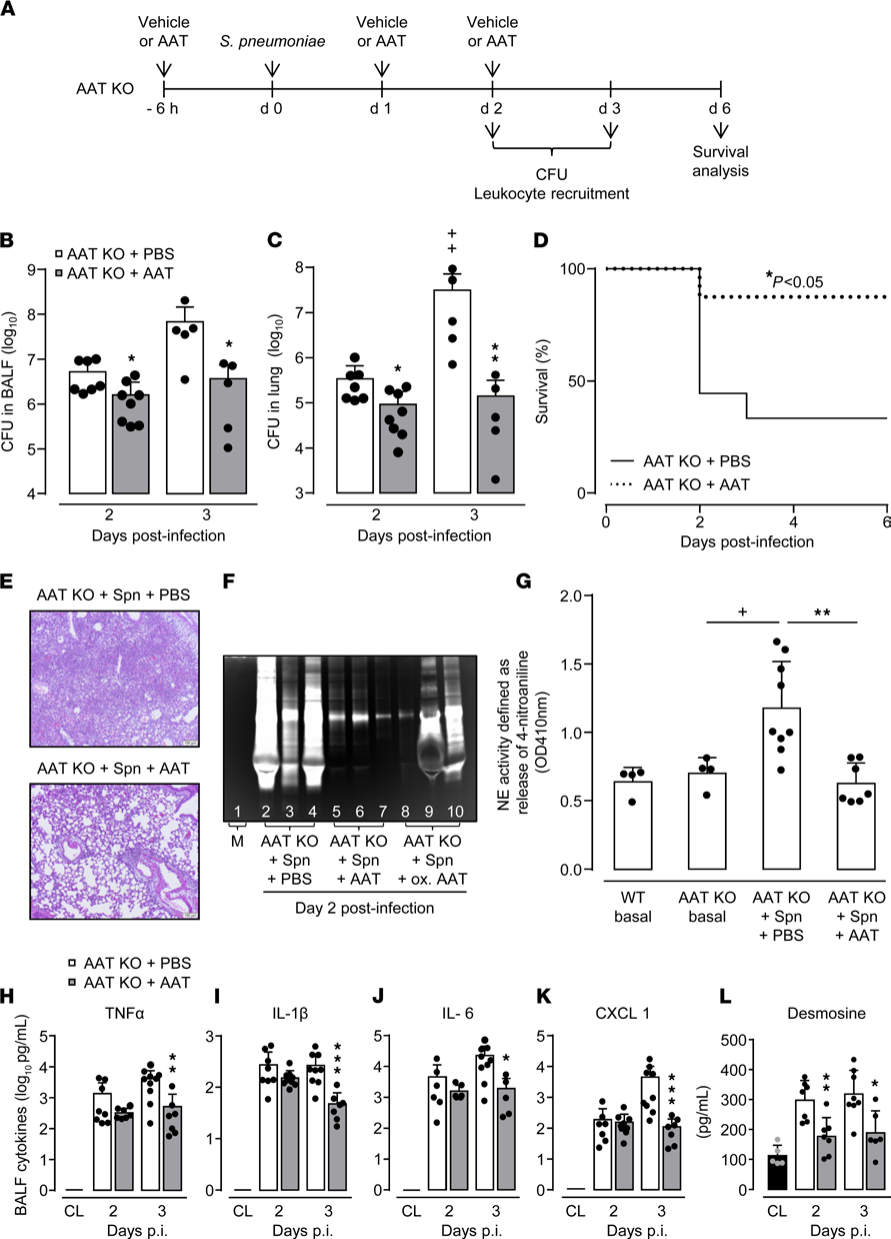
Effect of AAT augmentation therapy on outcome in *S*. *pneumoniae*–infected AAT-KO mice. (**A**) Experimental setup. AAT-KO mice were treated with Respreeza (gray bars) or vehicle i.p. (white bars), as indicated. (**B** and **C**) Bacterial load in BALF (**B**) and lung tissue (**C**) at 48 hours and 72 hours postinfection. Data are shown as mean ± SD of *n* = 5–8 mice per time point and group. (**D**) Survival of AAT as compared with vehicle-treated, *S*. *pneumoniae*–infected AAT-KO mice (*n* = 9 mice per group). (**E**) Lung histopathology of *S*. *pneumoniae*–infected AAT-KO mice after PBS (upper illustration) or hAAT augmentation therapy as outlined in **A** (lower illustration in **E**). The data are representative of *n* = 5 mice per group. Original magnification, x10. (**F**) Proteolytic activity in BALF of *S*. *pneumoniae*–infected AAT-KO mice receiving PBS or native or oxidized AAT. The data are representative of *n* = 5–6 mice per group. M, marker (gel without loaded sample). (**G**) NE activity in BAL neutrophils at day 1 postinfection. Bone marrow–derived neutrophils from untreated WT or AAT-KO mice served as baseline controls. Data are shown as mean ± SD of *n* = 4–9 mice per time point and treatment group. (**H**–**L**) TNF-α, IL-1β, IL-6, CXCL1, and desmosine levels in BAL fluids of mice at days 2–3 postinfection. Data are shown as mean ± SD of *n* = 7–9 mice per time point and treatment group. *P ≤ 0.05, **P ≤ 0.01, ***P ≤ 0.001 compared with vehicle-treated mice, ^+^*P* ≤ 0.05 compared with day 2 (Mann-Whitney *U* test, log-rank test). All data are representative of 2 independent experiments.

Based on the observation that AAT-KO mice were highly susceptible to pneumococcal pneumonia, we questioned what effect hAAT therapy would have on lung protective immunity in AAT-KO mice against *S. pneumoniae*. As shown in Figure 3, AAT-KO mice demonstrated a significantly improved bacterial clearance on days 2–3 postinfection after initiation of hAAT therapy, whereas bacterial outgrowth was observed in PBS-treated AAT-KO mice infected with *S. pneumoniae* (Figure 3, B and C). In line, AAT-KO mice receiving hAAT therapy had significantly improved survival of pneumococcal pneumonia compared with PBS-treated, *S. pneumoniae*–infected AAT-KO mice (Figure 3D). As expected, histopathology of lungs from PBS-treated, *S. pneumoniae*–infected AAT-KO mice revealed severe pneumococcal pneumonia, while lungs of *S. pneumoniae*–challenged AAT-KO mice treated with hAAT demonstrated substantially less severe interstitial and alveolar inflammation (Figure 3E).

We next examined the effect of hAAT therapy on total proteolytic and NE activity in BALF and BAL neutrophils of *S. pneumoniae*–infected mice of the 2 treatment groups. Using gelatin-based zymography (Figure 3F), we observed substantial proteolytic (i.e., gelatinolytic) activity in BALF of *S. pneumoniae*–infected AAT-KO mice (illustrated by white proteolysis bands in lanes 2–4), which was drastically blocked by the therapy with hAAT (lanes 5–7), and only partially blocked by oxidized AAT, which is known to be devoid of NE-inhibitory activity (27, 28) (lanes 8–10). In selected experiments, we also examined the effect of AAT therapy on BAL NE activity in mice. Baseline NE activity was observed in peripheral neutrophils from untreated WT and AAT-KO mice serving as controls (Figure 3G). Of note, BAL neutrophils of *S. pneumoniae*–challenged AAT-KO mice treated with hAAT showed significantly lower NE activity compared with *S. pneumoniae*–infected, PBS-treated AAT-KO mice, thus illustrating a link between systemic therapy with hAAT and inhibition of NE activity in BAL neutrophils of *S. pneumoniae*–challenged AAT-KO mice (Figure 3G). NE is a highly potent serine protease that is able to degrade lung elastin fibers and to activate proinflammatory cytokines (12, 13, 29). In line with this, we found significantly reduced levels of TNF-α, IL-1β, IL-6, CXCL1, and elastin-derived desmosine in BALF of *S. pneumoniae*–infected AAT-KO mice receiving therapy with hAAT (Figure 3, H–L). Notably, reported differences in outcome between groups were not due to differences in leukocyte subset recruitment (data not shown).

### Augmentation therapy improves lung antibacterial immunity in AAT-KO mice via inhibition of NE.

**Figure 4:**
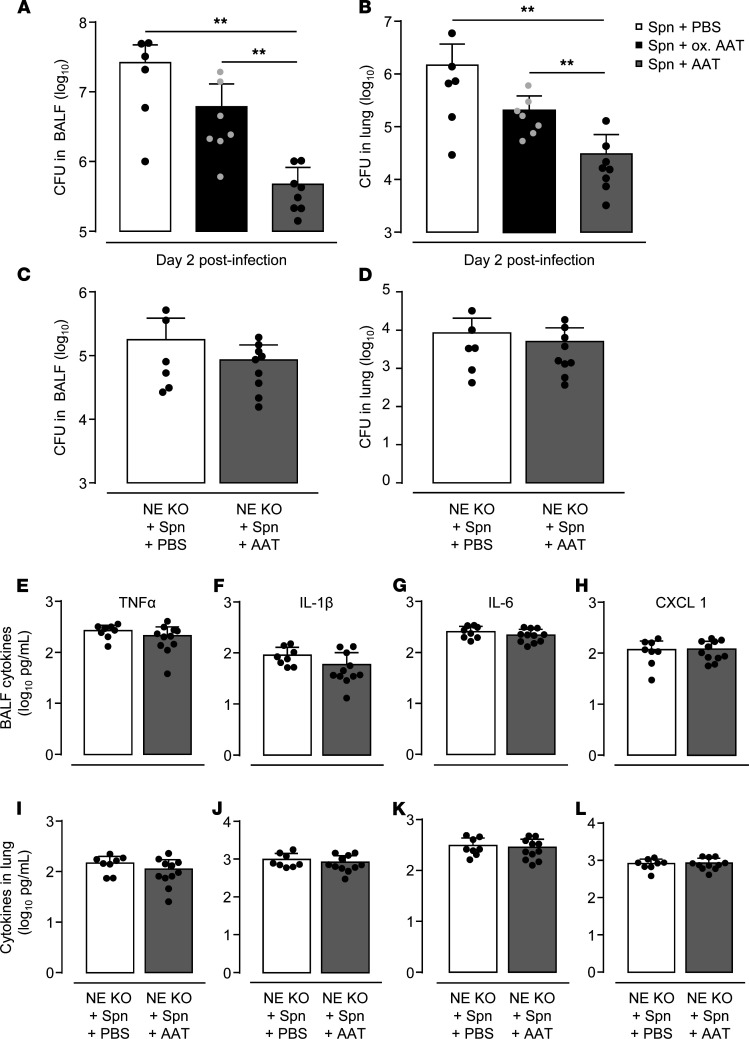
Effect of AAT augmentation therapy with active or oxidized AAT on outcome in *S*. *pneumoniae*–infected AAT-KO and NE-KO mice. AAT-KO mice were treated with Respreeza (gray bars), vehicle (white bars), or oxidized Respreeza (black bars) i.p. at 6 hours prior to pneumococcal infection and at days 0 and 1 postinfection. (**A** and **B**) Bacterial loads in BALF (**A**) and lung tissue (**B**) were determined at 48 hours postinfection. Data are shown as mean ± SD of *n* = 6–8 mice per time point and treatment group and are representative of 2 independently performed experiments. NE-KO mice were treated with Respreeza (gray bars) or vehicle (white bars) i.p. starting 24 hours postinfection with *S*. *pneumoniae*. (**C** and **D**) Bacterial loads in BALF (**C**) and lung tissue (**D**) were determined at 48 hours postinfection. Data are shown as mean ± SD of *n* = 6–9 mice per time point and treatment group and are representative of 2 independently performed experiments. (**E**–**L**) TNF-α, IL-1β, IL-6, and CXCL1 cytokine levels in BAL fluids and lung homogenate supernatants of NE-KO mice at day 2 postinfection, as indicated. Data are shown as mean ± SD of *n* = 8–11 mice per time point and treatment group and are representative of 2 independently performed experiments. **P ≤ 0.01, compared with vehicle-treated mice. (Mann-Whitney *U* test, log-rank test.)

To exclude potential immunomodulatory properties of hAAT therapy underlying the observed effects in *S. pneumoniae*–infected AAT-KO mice, we treated AAT-KO mice with oxidized or native hAAT followed by infection with *S. pneumoniae*. Of note, oxidation of the methionine residues of AAT substantially decreases its ability to inhibit various proteases, including the serine protease NE, while preserving its immunomodulatory activity (27, 28). AAT-KO mice augmented with hAAT demonstrated significantly improved bacterial clearance in BALF and lung tissue on day 2 postinfection, while no significant effect was observed in AAT-KO mice treated with oxidized hAAT (Figure 4, A and B). In additional experiments, *S. pneumoniae*–challenged NE-KO mice treated with vehicle or hAAT had similar CFU counts in their lungs (Figure 4, C and D). No significant differences in BALF cytokines were noted between groups (Figure 4, E–L). Thus, therapy with hAAT appears to improve lung protective immunity primarily by inhibition of proteolysis, specifically the activity of NE.

### Effect of AAT deficiency on bacterial phagocytosis, killing, and burst induction in professional phagocytes.

**Figure 5:**
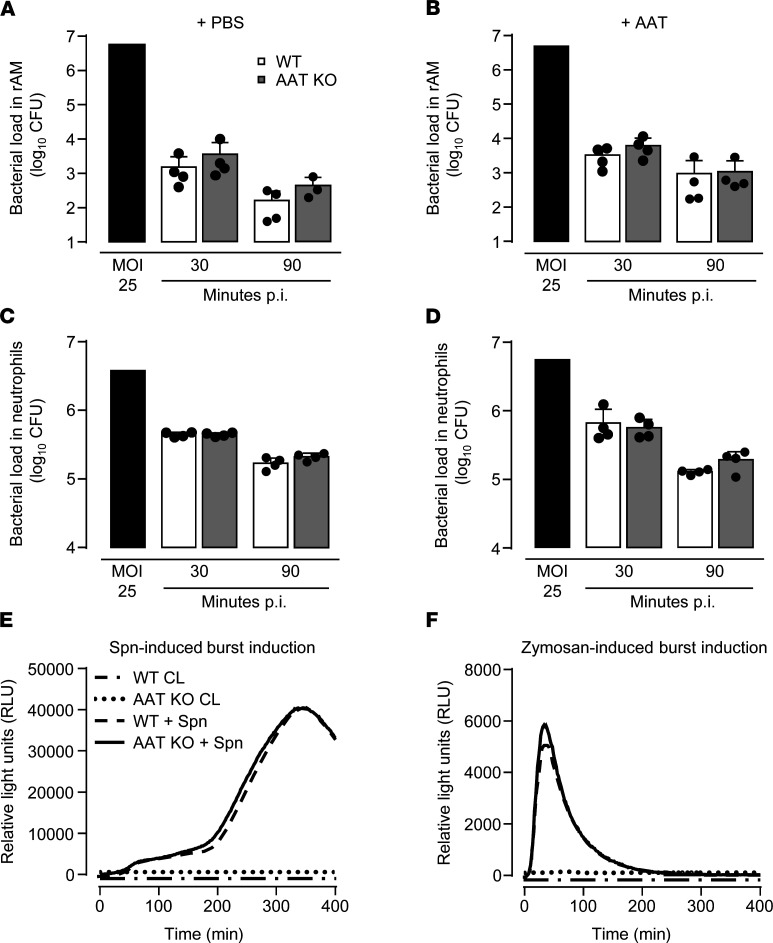
Phagocytosis, killing, and burst induction in *S*. *pneumoniae*-infected alveolar macrophages and bone marrow-derived neutrophils of WT and AAT-KO mice. (**A**–**D**) Phagocytosis capacity of resident alveolar macrophages (rAMs) and bone marrow–derived neutrophils (BM-PMNs) infected with *S*. *pneumoniae* was analyzed at 30 minutes postinfection of cells with *S*. *pneumoniae* at a multiplicity of infection (MOI) of 25. The killing capacity of rAMs and BM-PMNs infected with *S*. *pneumoniae* in the absence or presence of AAT was determined at 90 minutes postinfection. (**E** and **F**) Formation of reactive oxygen species (burst) in highly purified BM-PMNs induced by *S*. *pneumoniae* (MOI 5) (**E**) or zymosan (**F**). Data are representative of 2 independent experiments with *n* = 4 mice per time point and treatment group.

Based on the observation that hAAT-augmented AAT-KO mice coped better with pneumococcal challenge compared with vehicle-treated, *S. pneumoniae*–infected AAT-KO mice, we examined bacterial phagocytosis, killing, and burst induction in professional phagocytes of WT and AAT-KO mice in the absence or presence of hAAT. As shown in Figure 5, A–D, no differences in pneumococcal phagocytosis and killing were observed between rAMs or BM-PMNs of WT or AAT-KO mice, in either the absence or presence of hAAT. Moreover, BM-PMNs of WT and AAT-KO mice responded similarly to *S. pneumoniae*– or zymosan-induced respiratory burst (Figure 5, E and F). These data illustrate that differences in antibacterial responses between WT and AAT-KO mice are not due to differences in pneumococcal phagocytosis and killing by the respective professional phagocyte subsets.

### Human AAT remains biologically active in S. pneumoniae–infected AAT-KO mice.

**Figure 6:**
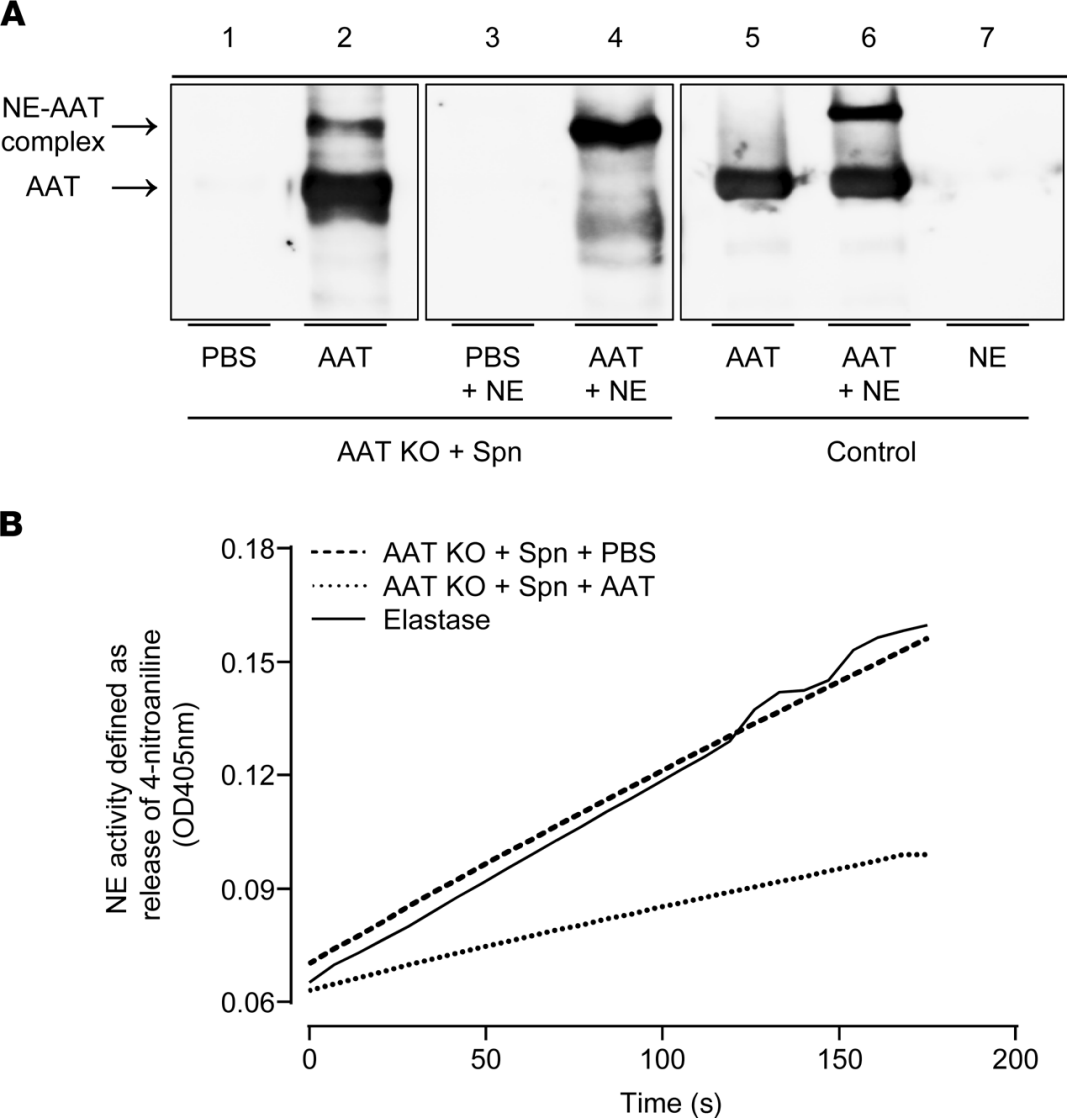
NE-AAT complex formation in BAL fluids of *S. pneumoniae*-challenged AAT-KO mice receiving augmentation therapy. (**A**) AAT (lane 2, lower protein band) and NE-AAT complex formation (lanes 2 and 4, upper protein band) detected in BAL fluids of AAT-augmented AAT-KO mice but not in PBS-treated AAT-KO mice (lanes 1 and 3). As a control, blotting of hAAT protein only gave rise to 1 single band in Western blot analysis (lane 5), and hAAT preincubated with purified NE in vitro resulted in 2 AAT-specific bands representing noncomplexed, native hAAT and NE-complexed hAAT (lane 6). NE protein alone gave no signal in AAT-specific Western blotting (lane 7). Lanes 1 to 7 were run on the same gel but were noncontiguous. (**B**) BAL fluids of *S*. *pneumoniae*–challenged AAT-KO mice receiving AAT augmentation therapy as compared with vehicle were subjected to kinetic NE activity assay. Purified elastase served as positive control. Data are representative of 3 independently performed experiments.

In the next set of experiments, we wished to determine whether hAAT employed in the current study would retain NE complex–forming activity during pneumococcal infection. NE-AAT complex formation was examined by using BALF of *S. pneumoniae*–challenged AAT-KO mice receiving hAAT therapy or vehicle. As shown in Figure 6A, BALF collected from PBS-treated, *S. pneumoniae*–challenged AAT-KO mice incubated or not with NE in vitro did not show any AAT protein in Western blots (Figure 6A, lanes 1, 3). However, AAT protein was easily detectable in BALF of *S. pneumoniae*–challenged AAT-KO mice treated with hAAT (lane 2), with 2 bands of different molecular size representing native AAT (lower band in lane 2) or NE-AAT complex (upper band in lane 2). Furthermore, the addition of purified NE to BALF of hAAT-augmented, *S. pneumoniae*–infected AAT-KO mice led to a nearly complete shift of the aforementioned lower band of native AAT to the protein band representing NE-AAT complexes (upper band in lane 4), clearly confirming the anti-NE activity of the hAAT preparation. As a control, blotting of hAAT protein gave a single band (lane 5), whereas hAAT preincubated with NE generated 2 AAT-specific bands of lower and higher molecular size representing native hAAT and NE-complexed hAAT, respectively (both lane 6). As expected, NE protein alone gave no signal in AAT-specific Western blotting (lane 7).

To confirm further that hAAT preserves anti-NE activity during pneumococcal pneumonia in AAT-KO mice, BALF of PBS-treated or hAAT-augmented, *S. pneumoniae*–challenged AAT-KO mice were subjected to kinetic NE activity assay. As shown in Figure 6B, BALF of PBS-treated and *S. pneumoniae*–infected AAT-KO mice exhibited easily detectable NE activity similar to that of purified NE (serving as positive control). However, AAT purified from BALF of hAAT-augmented, *S. pneumoniae*–challenged AAT-KO mice showed substantially less NE activity (Figure 6B). Hence, exogenously applied hAAT seems to be fully active during pneumococcal infection of AAT-KO mice and is not inactivated by any putative infection-evoked reactive intermediates.

### Attenuated antibacterial immunity in AAT-KO mice is due to NE-dependent degradation of alveolar collectins surfactant protein A and D.

**Figure 7:**
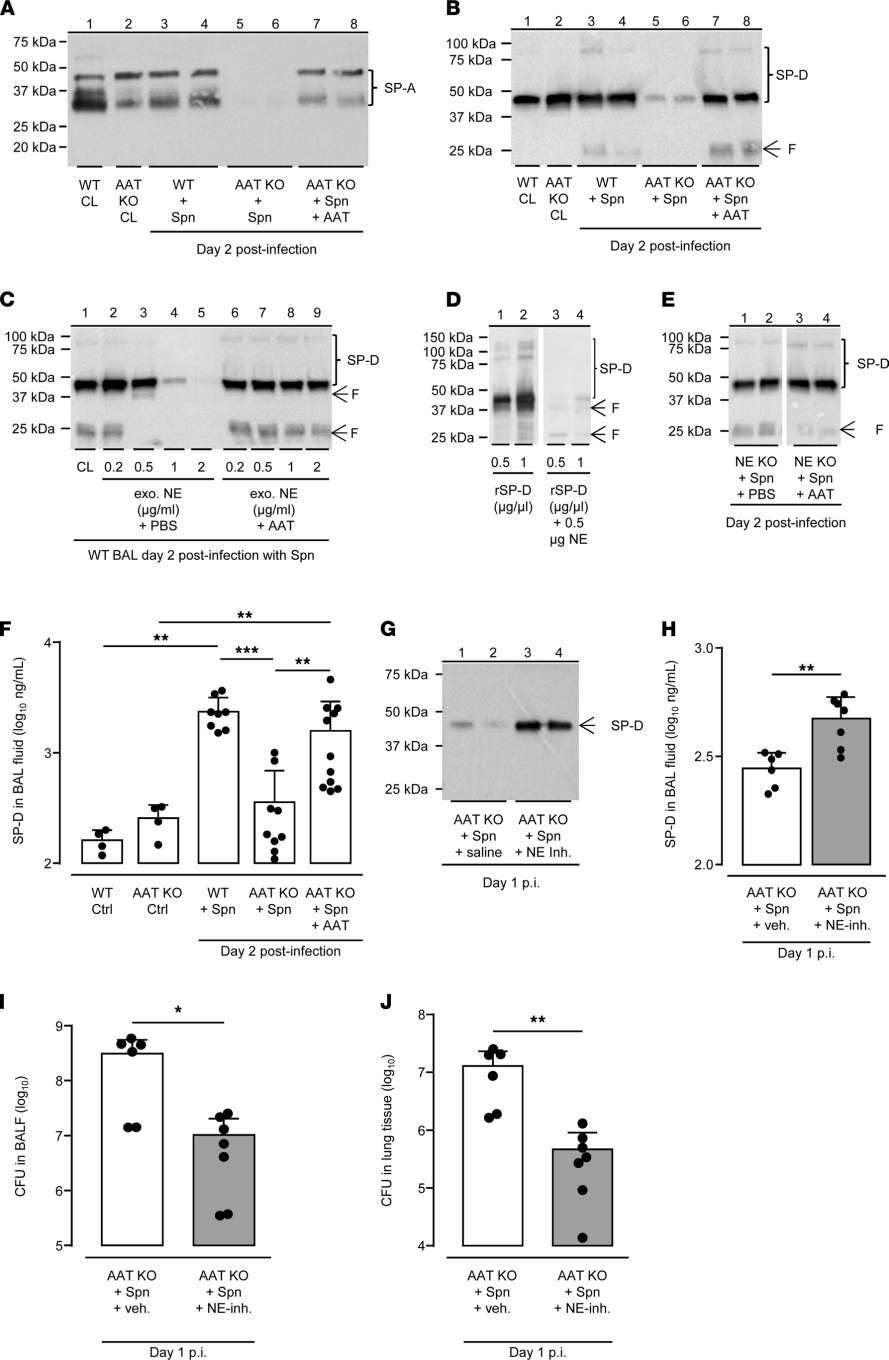
NE degrades collectins SP-A and SP-D in the lungs of AAT-deficient mice. (**A** and **B**) SP-A (**A**) and SP-D (**B**) proteins in BAL fluids of untreated or *S*. *pneumoniae*–infected WT mice (lanes 3, 4) as compared with AAT-KO mice (lanes 5, 6) as compared with AAT-augmented AAT-KO mice (lanes 7, 8) on day 2 postinfection (F, fragment). (**C**) Dose-dependent degradation of SP-D by NE in BAL fluids of *S*. *pneumoniae*–challenged WT mice (lanes 2–5), while preincubation of NE with AAT fully protected SP-D protein from degradation (lanes 6–9). (**D**) Incubation of recombinant SP-D protein with NE leads to degradation of recombinant SP-D protein. (**E**) SP-D protein in BAL fluids of *S*. *pneumoniae*–infected NE-KO mice with or without AAT augmentation therapy. (**F**) Quantification of SP-D protein levels in BAL fluids of mice of the respective treatment groups by ELISA, as indicated. (**G**–**J**) Effect of treatment of *S*. *pneumoniae*-challenged AAT-KO mice with selective NE inhibitor Sivelestat on SP-D levels in BAL fluids (**G** and **H**) and bacterial loads in BAL fluids (**I**) and lung tissue (**J**) of AAT-KO mice relative to vehicle treatment, as indicated. (**D** and **E**) Lanes 1 to 4 were run on the same gel but were noncontiguous. Data are shown as mean ± SD of *n* = 6–10 mice per time point and treatment group and are representative of 2 independently performed experiments. *P ≤ 0.05, **P ≤ 0.01, ***P ≤ 0.001, compared with vehicle-treated mice. (Mann-Whitney *U* test.)

Previous studies in patients with cystic fibrosis revealed that elastolytic activities in BAL fluids were inversely related to levels of respiratory opsonins (30). Therefore, we next questioned whether NE might contribute to the attenuated antibacterial responses in *S. pneumoniae*–challenged AAT-KO mice by functionally depleting alveolar collectins surfactant protein A (SP-A) and SP-D. As shown in Figure 7, WT and AAT-KO mice exhibited similar levels of alveolar SP-A and SP-D under baseline conditions (Figure 7, A and B, lanes 1, 2). In response to *S. pneumoniae* challenge, SP-A and SP-D proteins (both monomeric 42 kDa and higher-order oligomeric protein bands (>75 kDa) in Figure 7B were easily detectable in BALF of WT mice, whereas both collectins were nearly completely degraded in BALF of *S. pneumoniae*–challenged AAT-KO mice (both monomeric and oligomeric forms) (Figure 7, A and B, lanes 5, 6). Importantly, such depletion of SP-A and SP-D could be completely inhibited by hAAT treatment of *S. pneumoniae*–infected AAT-KO mice (Figure 7, A and B, lanes 7, 8).

We next aimed to clarify whether NE was indeed responsible for the observed SP-A/SP-D degradation in *S. pneumoniae*–challenged AAT-KO mice. Therefore, we examined the effect of increasing concentrations of purified NE on SP-D protein levels in BALF of *S. pneumoniae*–challenged WT mice. As shown in Figure 7C, we found that NE even at a concentration of 0.5 μg was sufficient to degrade SP-D protein into even smaller SP-D fragments (lane 3), while addition of 2 μg NE per sample completely degraded SP-D protein in BAL fluids of *S. pneumoniae*–challenged WT mice (Figure 7C, lane 5). Importantly, preincubation of purified NE with hAAT in vitro (in order to complex and thus inactivate the protease prior to addition to BALF) completely protected BALF-derived SP-D proteins from the degradation (Figure 7C, lanes 6 to 9).

In the next set of experiments, we examined whether purified NE would also degrade recombinant SP-D (rSP-D) protein in vitro. Of note, addition of just 0.5 μg purified NE per sample was sufficient to nearly completely degrade rSP-D protein in vitro (Figure 7D, lane 3 and 4), similar to the observation made in BAL fluids of WT mice (Figure 7C).

We next hypothesized that if NE is the culprit to degrade collectins SP-A and SP-D both in vivo and in vitro, NE-deficient mice should be protected from NE-mediated depletion of alveolar collectins. Indeed, as shown in Figure 7E, SP-D protein was easily detectable in BALF of *S. pneumoniae*–challenged NE-KO mice, with similar band intensities observed in *S. pneumoniae*–challenged WT mice (compare lanes 1, 2 in Figure 7E with lanes 3, 4 in Figure 7B), and a similar pattern of SP-D was observed in AAT-treated, *S. pneumoniae*–challenged NE-KO mice. These data clearly support our hypothesis that neutrophil-derived NE is the major protease mediating degradation of lung collectins in *S. pneumoniae*–infected AAT-KO mice.

Using a sensitive ELISA for quantification of alveolar SP-D, we found that SP-D levels significantly increased in BALF of WT mice upon pneumococcal challenge compared with untreated WT mice, whereas upon *S. pneumoniae* challenge BALF SP-D levels of AAT-KO mice increased only weakly and nonsignificantly (Figure 7F). Importantly, the infection-driven rise in SP-D levels could be significantly restored in BALF of AAT-KO mice upon therapy with hAAT (Figure 7F).

Finally, we hypothesized that if NE-mediated degradation of lung collectins contributes to the disturbed antibacterial immunity in AAT-KO mice, blockade of NE with specific inhibitors should reverse this process. To this end, AAT-KO mice were treated with the highly specific NE inhibitor Sivelestat twice daily subsequent to *S. pneumoniae* infection. As shown in Figure 7G, therapeutic application of Sivelestat protected SP-D from NE-dependent degradation in BALF of *S. pneumoniae*–infected AAT-KO mice, relative to vehicle-treated, *S. pneumoniae*–challenged AAT-KO mice completely lacking alveolar SP-D protein and when compared with *S. pneumoniae*–challenged WT mice (compare lanes 3, 4 in Figure 7G with lanes 3, 4 in Figure 7B). Again, quantification of SP-D levels by ELISA confirmed significantly increased SP-D levels in BALF of *S. pneumoniae*–infected AAT-KO mice after treatment with Sivelestat, reaching a similar order of magnitude as observed in AAT-treated, *S. pneumoniae*–infected AAT-KO mice (compare Figure 7H with Figure 7F). Most importantly, we found that treatment with Sivelestat inhibited exaggerated NE activity, and thus loss of alveolar collectins led to significantly decreased bacterial loads both in BAL fluids and lungs of *S. pneumoniae*–infected AAT-KO mice (Figure 7, I and J).

Collectively, our data show that NE is the major culprit in degrading SP-A and SP-D in *S. pneumoniae*–challenged AAT-KO mice, thereby disturbing lung protective immunity in AAT-deficient mice. The latter can be restored by both hAAT augmentation therapy and using synthetic NE inhibitors.

## Discussion

In the current study, we evaluated the impact of AATD on lung protective immunity against *S*. *pneumoniae* in mice in the absence or presence of AAT augmentation therapy. To reflect the clinical situation of patients with inherited AATD, we used recently generated AAT-KO mice lacking all 5 mouse serpin A1 paralogues, thus leading to complete AAT protein deficiency. AAT-KO mice were used at the age of 8–14 weeks while spontaneously developing lung emphysema only at advanced age of more than 35 weeks (25). Based on the data generated from this model, we show that AATD is detrimental to mice infected with *S*. *pneumoniae* but could be rescued upon treatment with plasma-purified hAAT.

Patients with AATD receive augmentation therapy primarily to slow down emphysema progression. To this end, patients with AATD typically receive standard doses of 60 mg/kg/wk intravenously to restore at least 50% of normal AAT serum levels (approximately 11 μM) (10). Although calculated standard doses may be sufficient to reverse the biochemical AAT defects in stable patients (10), little is known about serum AAT levels needed in case of microbial challenge where AAT turnover kinetics might be increased, as observed in the current study. Actually, previous studies showed that patients with AATD are more likely to suffer from pulmonary infections, which could be ameliorated by AAT therapy. A recent study by Balbi et al. reported that augmentation therapy in AATD patients with COPD significantly reduced the percentage of gram-positive and -negative bacteria (*H*. *influenzae*, *S*. *pneumoniae*) compared with controls (23). In addition, in a (subjective) questionnaire-based study by Lieberman, patients with ZZ AATD reported significantly fewer lung infections per year under augmentation therapy compared with those subjects with the same phenotype who were not receiving this therapy (31). Moreover, cystic fibrosis (CF) patients with deficient variants of AAT demonstrated an earlier carriage of *P*. *aeruginosa*, which was correlated with an earlier onset of *P*. *aeruginosa* lung infection (32). In contrast, CF patients receiving AAT therapy demonstrated significantly lower bacterial loads of *P*. *aeruginosa* (33). Together, these studies strongly support the view that AATD is associated with attenuated antibacterial immunity and that augmentation therapy contributes to the reduced incidence and severity of lung bacterial colonization and/or infection in AATD but also in non-AATD CF patients.

Lower elastolytic activities were reported in BALF of patients with AATD under standard augmentation therapy (19). In our experimental settings, bacterial infection significantly decreased plasma AAT levels in mice by approximately 25% (Figure 1), allowing us to predict that bacterial infection in patients with AATD would result in a transient drop of AAT plasma levels. Thus, the currently applied AAT therapy dosing regimen may be sufficient to diminish bacterial burden in patients with AATD but may be too low to adequately neutralize increasing NE activity due to the peripheral neutrophilia developing in response to acute bacterial infection. Based on a previous study (19) and our report, clinically relevant arguments to increase AAT augmentation therapy to more physiological levels may be (a) to facilitate an improved inhibition of ECM-degrading NE activity in the bronchoalveolar space of AATD patients (19) and (b) to better protect AATD patients against increased AAT turnover expectedly developing upon lung bacterial infections, which would also lower the risk of emphysema progression/exacerbation.

Various aspects may explain the observed infection-induced decrease of plasma AAT levels in mice. First, both elevated AAT consumption caused by peripheral neutrophilia and/or leakage of AAT into the alveolar space may have contributed to the observed drop in AAT protein levels. Moreover, an active trans-endo-/epithelial transport of AAT has also been described (34, 35). Third, it is conceivable that septic liver injury developing in response to invasive pneumococcal disease may also result in decreased AAT serum levels, although this aspect is unlikely to be relevant in the current model of focal pneumonia.

AAT-KO mice were found to succumb to pneumococcal lung infection by day 2 postinfection. We found a markedly higher NE activity in BAL-derived neutrophils of *S. pneumoniae*–infected AAT-KO as compared with WT mice. This finding is in line with the lack of antiprotease activity of AAT in the KO but not in the WT mice, which actually responded to pneumococcal lung infection with significantly increased bronchoalveolar release of AAT. NE is a highly potent serine protease able to degrade lung ECM, such as elastin, and may facilitate proinflammatory cytokine activation in the inflammatory microenvironment (12, 13, 29). In the current study, we show that levels of desmosine, an amino acid released from elastin upon its degradation, increased substantially in BALF of *S. pneumoniae*–challenged AAT-KO relative to WT mice. These data demonstrate that NE actually triggers ECM degradation in the lungs of mice in the absence of AAT, and this process is profoundly inhibited upon AAT augmentation. Furthermore, analysis of the proinflammatory cytokines TNF-α, IL-1β, IL-6, and CXCL1 illustrated the effect of AAT augmentation therapy on lung inflammatory responses after bacterial challenge in nonaugmented as compared with augmented AAT-KO mice. Actually, these data show that augmentation therapy of *S. pneumoniae*–infected AAT-KO mice improved survival by effectively diminishing lung bacterial loads and the release of proinflammatory cytokines, which — in addition to the bacterial infection — may significantly contribute to lung damage. Together, these data clearly show an important role of alveolar AAT in blocking NE-mediated lung tissue damage in mice. Our findings are consistent with similar observations in AATD patients subjected to AAT augmentation therapy (19), thus supporting the clinical relevance of the current AAT-KO mouse model of AATD.

To explain the observed differences in antibacterial responses discriminating *S. pneumoniae*–challenged WT from AAT-KO mice, we examined the bacterial phagocytosis, killing, and burst capacity of alveolar macrophages and neutrophils from WT and AAT-KO mice in vitro. However, we did not note any significant differences between groups. Hence, it seems very unlikely that the observed differences in bacterial loads between WT and AAT-KO mice are due to principal differences in the phagocytosis/killing or respiratory burst capacity of professional phagocytes between these 2 experimental groups.

However, based on the observation that AAT-KO mice exhibited a drastically increased proteolytic burden in their lungs upon pneumococcal challenge, which was largely blocked by AAT therapy along with a significant reduction in lung bacterial loads, we hypothesized that NE was the major culprit to disturb lung antibacterial immunity in *S. pneumoniae*–challenged AAT KO. Previous studies in patients with CF showed that NE is capable of disturbing lung antibacterial immunity at different levels, including degradation of the opsonophagocytically important collectins SP-A and -D (36, 37), as well as degradation of pathogen-specific immunoglobulins (30). According to our understanding, pathogen-evoked protective antibody responses were very unlikely to develop so quickly in our employed acute lung infection model. However, the hydrophilic lung surfactant proteins SP-A and SP-D are well-known to serve as opsonophagocytically relevant collectins to orchestrate lung host defense against various lung-tropic pathogens, including *S*. *pneumoniae*, based on in vitro studies and genetically engineered animal models (38–43). In the current study, we provide evidence that NE is responsible for the observed degradation of SP-A and SP-D in *S*. *pneumoniae*–infected AAT-KO mice, which is accompanied by decreased antibacterial defense. Of note, in addition to the currently reported degradation of lung collectins by neutrophil-derived NE, peroxynitrite may also affect the binding activity of SP-A and SP-D, due to its strong nitrating and oxidizing effects (44). Other studies reported neutrophil-derived myeloperoxidase-dependent inactivation of SP-D in vivo (45). Actually, our findings provide a potentially novel and hitherto unrecognized molecular mechanism by which inherited AATD affects lung antibacterial immunity, besides its well-known effect on lung emphysema progression in patients with AATD, and how this defect can be corrected by augmentation therapy.

Chronic lung diseases like CF or COPD are characterized by a vicious circle of bronchoalveolar recruitment of neutrophils with subsequent excess release of NE culminating in end-stage emphysematous lung injury. At the same time, both CF and COPD patients have increased susceptibility to opportunistic bacterial infections, which at least in CF is causally responsible for excess lung neutrophil recruitment. A previous study suggested particularly NE to be responsible for mediating lung collectin depletion in BAL fluids of CF patients (46), although this study did not relate these findings to antibacterial immune defects. As mentioned before, a recent study by Balbi et al. (23) reported that AAT treatment significantly reduced total bacterial loads, including *S*. *pneumoniae*, in sputum samples of COPD patients when compared with untreated COPD patients. Therefore, when taking together previous studies and our work, it appears reasonable that the success of AAT augmentation therapy of (mostly neutrophil-dominated) chronic lung diseases should not only rely on long-term inhibition of emphysema development or progression but also should aim at inhibition of NE-dependent depletion of SP-A and SP-D in order to avoid defective antibacterial responses.

Collectively, we propose the following model: in WT mice, NE released into the alveolar airspace upon infection (at least partially) becomes complexed with AAT, thereby limiting both NE-driven degradation of lung ECM components as well as lung collectins SP-A and SP-D. In contrast, in AAT-KO mice, *S. pneumoniae* challenge triggers increased alveolar neutrophil accumulation and thus NE liberation that is not inhibited by AAT. This consequently triggers increased NE-dependent lung ECM degradation, as determined by increased alveolar desmosine levels, and even further triggers a nearly complete functional depletion of SP-A and SP-D collectins within the alveolar compartment, thereby profoundly affecting lung bacterial pathogen elimination. Upon AAT augmentation therapy or application of NE-specific inhibitors, excessive neutrophil-derived NE activity is blocked, thus inhibiting ECM degradation and degradation of collectins, thereby facilitating a normalized bacterial pathogen elimination process similar to that observed in *S. pneumoniae*–challenged, AAT-sufficient WT mice.

As such, the current study expands our understanding of the impact of augmentation therapy on lung protective immunity in patients with inherited AATD and how this therapy might be combined in the future with more cost-efficient NE-specific inhibitor therapies. Importantly, however, we believe that these data may also be critically important to other neutrophil-dominated lung diseases where continuous recruitment of neutrophils is associated with defects in lung antibacterial immunity, including (but not limited to) CF, COPD, as well as bronchiectasis.

## Methods

### Animals.

WT mice (C57BL/6) were purchased from Janvier Lab (Le Genest-Saint-Isle, France). AAT-KO mice lacking all of the 5 serpin A1 a-e paralogues expressed in mice were generated as described recently (25). NE-KO mice were purchased from The Jackson Laboratory (Bar Harbor, Maine, USA) (47). Age- and sex-matched mice were used for experiments at 8 to 14 weeks of age.

### Reagents.

Respreeza (plasma-purified human AAT) was purchased from CSL Behring (Marburg, Germany). NE-specific inhibitor Sivelestat (48) was purchased from MilliporeSigma (St. Louis, Missouri, USA). Anti-CD45 PE-Cy7 (clone 30-F11) and anti-Ly6G V450 (clone 1A8) were purchased from BD Biosciences (Heidelberg, Germany). Mouse neutrophil isolation kit was purchased from MACS, Miltenyi Biotec (Bergisch Gladbach, Germany). For Western blot analysis, polyclonal rabbit anti-mouse SP-A antibody (catalog ABIN3043205) was purchased from antibodies-online (Aachen, Germany). Monoclonal rabbit anti-mouse SP-D antibody (clone EPR21774-143) and monoclonal rabbit anti-hAAT antibody (clone EPR17087-50) were purchased from Abcam (Cambridge, United Kingdom). rSP-D was purchased from antibodies-online. Antigen specificity of the employed anti-hAAT antibody, or anti-mouse SP-A and anti-mouse SP-D antibody, was confirmed by liquid chromatography-mass spectrometry at the Proteomics Core facility of Hannover Medical School (data not shown).

### Culture and quantification of S. pneumoniae.

For details, see Supplemental Methods.

### Growth kinetics of S. pneumoniae in the presence of AAT.

For details, see Supplemental Methods.

### Infection of mice with S. pneumoniae.

Infection of mice with capsular group 19F *S*. *pneumoniae* was done as recently described (49, 50). For further information, see Supplemental Methods.

### Determination of bacterial loads in BAL fluids and lung homogenates.

For details, see Supplemental Methods.

### Oxidation of human purified AAT.

Oxidization of native hAAT was achieved as described previously (27, 28, 51, 52).

### Treatment groups.

Five treatment groups were established: WT mice were mock infected (PBS) or were infected with *S. pneumoniae* as described above, and endogenous AAT levels were determined in plasma and BALF at different time points postinfection.

The role of AAT deletion on evolving pneumococcal pneumonia was examined in AAT-KO mice and WT mice (serving as controls), which were infected orotracheally with *S. pneumoniae*, and disease progression was monitored during an observation period of 6 days.

The effect of AAT augmentation on antibacterial immunity in AAT-KO mice was studied by treating AAT-KO mice i.p. with either purified hAAT (2 mg/mouse twice daily) or PBS (vehicle control) at 6 hours prior to infection with *S. pneumoniae* and subsequent determination of CFU in BALF and lung tissue as well as assessment of survival and lung histopathology.

To exclude potential immunomodulatory effects of AAT on lung protective immunity against *S. pneumoniae* in mice, AAT-KO mice were treated i.p. with PBS, purified hAAT (exhibiting NE-inhibitory and putative immunomodulatory activity) (2 mg/mouse twice daily), or oxidized hAAT lacking NE-inhibitory activity (2 mg/mouse twice daily) (27, 28, 51, 53) followed by infection with *S. pneumoniae*. In additional experiments, NE-deficient mice were treated with human purified AAT or PBS starting at 24 hours postinfection followed by determination of bacterial loads and cytokine responses in BALF and lung tissue.

To analyze the effect of selective NE inhibition on lung antibacterial immunity in AAT-KO mice, mice were treated i.p. with Sivelestat (2 mg/mouse twice daily) or saline (vehicle control), starting immediately after infection with *S*. *pneumoniae*. Bacterial loads in BALF and lung tissue, as well as SP-D levels in BALF of *S. pneumoniae*–challenged AAT-KO mice of both treatment groups, were determined on day 1 postinfection.

### Phagocytosis and bacterial killing assay.

Phagocytosis and bacterial killing assays were performed as recently described (54–56). For further details, see Supplemental Methods.

### Analysis of respiratory burst induction in BM-PMNs.

For details, see Supplemental Methods.

### Isolation of AAT protein from BAL fluids of S. pneumoniae–infected mice.

AAT protein was purified from BALF of mice by affinity chromatography using AAT-specific Alpha-1 Antitrypsin Select matrix (Cytiva, Marlborough, Massachusetts, USA) according to the manufacturer’s recommendations. In brief, pooled BAL samples were diluted 1:3 with binding buffer (20 mM Tris/HCl 150 mM NaCl pH 7.4) and loaded onto Alpha-1 Antitrypsin Select columns. AAT was eluted in elution buffer (2 M MgCl_2_ in 20 mM Tris/HCl pH 7.6), and AAT concentrations were measured via NanoDrop 1000 Spectrophotometer (Thermo Fisher Scientific, Waltham, Massachusetts, USA). AAT-containing fractions were concentrated and buffer was changed to PBS using 10 kDa cutoff centricons (MilliporeSigma, Burlington, Massachusetts, USA).

### Western blot analysis.

Plasma and BALF samples of untreated or *S*. *pneumoniae*–infected WT and AAT-KO mice treated with PBS or hAAT were subjected to SDS-PAGE under reducing conditions followed by Western blot analysis of hAAT protein or collectins SP-A and SP-D, according to previously published protocols (49, 54). Exogenously applied hAAT was detected in mice using anti-hAAT antibody. Collectins SP-A and SP-D were detected using rabbit anti-mouse anti-SP-A antibody or rabbit anti-mouse anti-SP-D antibody. Protein bands were detected by enhanced chemiluminescence signals (ECL Plus; Cytiva, Buckinghamshire, United Kingdom) using a Vilber Lourmat Chemismart 5000 analyzer and Bio1D software (Vilber Lourmat, Eberhardzell, Germany).

For NE-dependent SP-D degradation experiments in vitro, indicated amounts of purified human NE were incubated with BALF of *S. pneumoniae*–infected WT mice or with recombinant murine SP-D protein (antibodies-online) for 15 minutes at 37°C. Inhibition of NE was done by incubating indicated amounts of purified NE with hAAT at a molar ratio of 1:2 for 1 hour at 37°C before adding NE-AAT complexes to the respective samples. Reactions were stopped by adding 4× SDS sample buffer (Bio-Rad Laboratories, Irvine, California, USA) with β-mercaptoethanol and heating at 95°C for 5 minutes.

Detection of NE complex formation with hAAT purified from BALF of hAAT-augmented mice was achieved by preincubating BALF-derived hAAT with either PBS or NE (5 ng/mL; MilliporeSigma, St. Louis, Missouri, USA) for 1 hour at 37°C. The reaction was stopped by adding equal volumes of 2× SDS sample buffer without β-mercaptoethanol and heating at 95°C for 3 minutes. Samples were then subjected to SDS-PAGE under nonreducing conditions followed by Western blot analysis of NE-hAAT complexes. Immune complexes were detected using a polyclonal rabbit anti-hAAT-specific antibody (Dako, Glostrup, Denmark) using ECL-enhanced chemiluminescence analysis.

### Gelatin-based zymography of total proteolytic activity in BAL fluids of S. pneumoniae–infected AAT-KO mice.

The total proteolytic activity in BAL fluids of *S. pneumoniae*–infected AAT-KO mice treated with PBS, native AAT, or oxidized AAT was determined by gelatin-based Novex Zymogram Plus gels (Thermo Fisher Scientific, Waltham, Massachusetts, USA). Briefly, BAL samples were diluted according to manufacturer’s instructions, equal volumes of 5 μL per well were loaded onto the gelatin-containing gel, and electrophoresis was performed as recommended by the manufacturer. Subsequently, gels were stained with Coomassie blue. Those regions of the gel, where the gelatin was degraded by active BAL fluid proteases, do appear as white bands on a black background.

### NE activity assay.

The effect of hAAT augmentation therapy on neutrophil-dependent elastase activity in *S. pneumoniae*–infected AAT-KO mice was examined by NE enzyme activity assay, according to previously published protocols (57–61). Briefly, BAL cells of *S. pneumoniae*–infected mice containing more than 90% neutrophils or BM-PMNs were centrifuged for 9 minutes at 430*g* at 4°C followed by lysis (5 minutes) of red blood cells using hypotonic lysis solution (AppliChem, Darmstadt, Germany) and subsequent washing in PBS. Neutrophil pellets were then washed once in ice-cold HBSS, and 2 × 10^6^ neutrophils per mouse were then transferred into small reaction tubes and centrifuged for 10 minutes at 600*g* at 4°C. Resulting neutrophil pellets were resuspended in 50 μL Tris buffer (0.2 M Tris, 0.15 M NaCl, 0.02 M CaCl_2_, pH 8.5) containing 20 μg/mL gentamicin (MilliporeSigma, St. Louis, Missouri, USA). For determination of NE activity, a standard curve ranging from 0 to 150 ng of catalytically active human NE/μL was established using commercially available human NE (MilliporeSigma, Burlington, Massachusetts, USA). NE-specific substrate MeO-Suc-Ala-Ala-Pro-Val-pNA (MilliporeSigma, St. Louis, Missouri, USA) was added to respective samples and standards in 96-well plates, which were then incubated for 1 hour at 37°C followed by measurements of NE activities expressed as release of 4-nitroaniline using a microplate reader operated at OD410 nm (VersaMax, Molecular Devices, Biberach, Germany). BM-PMNs from WT and AAT-KO mice served as baseline controls, and alveolar recruited neutrophils from *S. pneumoniae*–infected, hAAT-treated NE-KO mice as well as neutrophils from *S. pneumoniae*–infected mice preincubated with hAAT in vitro served as negative controls.

For kinetic NE activity measurements, samples were transferred to 96-well plates, purified elastase (stock solution 20 μg/mL, MilliporeSigma, St. Louis, Missouri, USA) and 0.1 M Tris buffer (pH 8.0) were added, and reaction mixtures were incubated at 37°C for 5 minutes. Following incubation, NE-specific substrate N-Succinyl-Ala-Ala-Ala-p-nitroanilide (MilliporeSigma, St. Louis, Missouri, USA) was added to the reaction mixtures. The absorbance was determined by using a microplate reader operated at OD 405 nm in a kinetic mode.

### Lung histopathology.

For details, see Supplemental Methods.

### Quantification of neutrophils in BAL and lung tissue.

For details, see Supplemental Methods.

### ELISA.

Murine AAT levels were measured in plasma and BALF of *S. pneumoniae*–infected mice using a commercially available mouse alpha-1 antitrypsin ELISA Kit (Crystal Chem Europe, Zaandam, Netherlands). Exogenously applied hAAT was measured in plasma and BALF of AAT-KO mice using a human Serpin A1 DuoSet ELISA lacking cross-reactivity for murine Serpin A1 (R&D Systems, Bio-Techne, Wiesbaden, Germany), according to the manufacturer’s instructions. TNF-α, IL-1β, IL-6, and CXCL1 cytokines were measured in BALF and lung homogenate supernatants of *S. pneumoniae*–infected AAT-KO mice and NE-KO mice receiving AAT augmentation therapy or vehicle using commercially available murine Quantikine ELISA Kits (R&D Systems, Bio-Techne). Mouse SP-D and desmosine levels were measured in BALF of untreated and *S. pneumoniae*–infected WT and AAT-KO mice using commercially available mouse SP-D (Abcam, Cambridge, United Kingdom) and mouse desmosine ELISA (Cusabio, Texas, USA) according to the manufacturers’ instructions.

### Statistics.

Statistical analysis was performed with GraphPad Prism software. Data are shown as mean ± SD values. Statistically significant differences between groups were analyzed by Mann-Whitney *U* test, and differences in survival between groups were analyzed using log-rank test. *P* values of less than 0.05 were considered statistically significant.

### Study approval.

All animal experiments corresponded to the European guideline 2010/63/EU and were authorized by the Lower Saxony State Office for Consumer Protection and Food Safety, Wardenburg, Germany.

## Author contributions

LO performed experiments, analyzed the data, and wrote the manuscript; RM, JS, LS, KK, and ST performed experiments; AP performed experiments and analyzed the data; CM provided material and discussed the data; and TW, SJ, and UAM designed the study, analyzed the data, and wrote the manuscript.

## Supplementary Material

Supplemental data

## Version 1 02/08/2021

Electronic publication
