# Supplementary material for: Alpha-1 antitrypsin deficiency impairs lung antibacterial immunity in mice
Source: JCI Insight. 2021 Feb 8;6(3):e140816. doi: 10.1172/jci.insight.140816 (PMC7934856; doi:10.1172/jci.insight.140816)
Supplement: Supplemental data [file jciinsight-6-140816-s056.pdf]

Online Data supplement

Alpha-1 antitrypsin deficiency impairs lung antibacterial immunity in mice

Lena Ostermann, Regina Maus, Jennifer Stolper, Lisanne Schütte, Konstantina Katsarou, Srinu Tumpara, Andreas Pich, Christian Mueller, Sabina Janciauskiene, Tobias Welte, Ulrich A. Maus

Material and Methods

Culture and quantification of S. pneumoniae

Culture and quantification of *S. pneumoniae* was done as previously described (1). Shortly, Todd Hewitt broth (THB, Sigma-Aldrich, St. Louis, USA) containing 20 % fetal bovine serum (FCS, Gibco, Thermo Fisher Scientific, Massachusetts, USA) was inoculated with type 19F *S. pneumoniae* which is known to cause focal pneumonia in mice (2, 3). Bacteria were grown to mid-log phase, then aliquoted and snap frozen, followed by storage at -80° C. For quantification, aliquots were thawed and ten-fold serial dilutions were plated onto sheep blood agar plates (BD Biosciences, Heidelberg, Germany) and incubated for 18 hours at 37° C, 5 % CO_2 followed by enumeration of colony forming units (CFU), and this procedure was repeated immediately prior to infection of mice to accurately determine applied infection doses.

Growth kinetics of S. pneumoniae in the presence of AAT

Growth kinetics of Spn in the presence of AAT in vitro were assessed in culture media supplemented with 20 % FCS in the presence of increasing concentrations of purified human Alpha-1 Antitrypsin (hAAT, Respreeza®, CSL Behring GmbH, Marburg, Germany), or PBS serving as control. At defined time points, aliquots of culture supernatants were plated in tenfold serial dilution onto sheep blood agar plates for subsequent determination of bacterial CFU, as described before.

Infection of mice with S. pneumoniae

Mice were infected with $1x10^7$ CFU Spn as described elsewhere (1, 4). Briefly, mice were anesthetized with tetrazoline hydrochloride (5 mg/kg) and ketamine (75 mg/kg). Then, a 26-gauge Abbocath catheter (Abbott, Wiesbaden, Germany) was placed into the trachea and a 1 ml syringe was connected to the catheter. Subsequently, the

bacterial suspension (in 50 µl PBS) was slowly aspirated into the lungs of mice under visual control. Anesthetized mock-infected WT mice received instillations of 50 µl PBS only. Afterwards, mice were brought back to their cages with free access to food and water and were monitored twice daily for disease symptoms.

Determination of bacterial loads in BAL fluids and lung homogenates

Bacterial loads were quantified in BAL fluids (BALF) and lung homogenates of Spn infected mice as reported previously (1, 5). Briefly, lungs were flushed repeatedly with small volumes of ice-cold phosphate-buffered saline (PBS) supplemented with 2 mM EDTA (Versen; Biochrom, Berlin, Germany) until a final volume of 6 ml was reached. Lungs were homogenized in 2 ml Hank's Balanced Salt Solution (HBSS, Biochrom GmbH, Berlin, Germany) and then filtered over a 100 µM cell strainer (Greiner, Frickenhausen, Germany) followed by determination of CFU.

Phagocytosis and bacterial killing assay

Phagocytosis and killing of *S. pneumoniae* were analyzed as described before (5-7). In brief, resident alveolar macrophages (rAM) were isolated from untreated WT and AAT KO mice by bronchoalveolar lavage. Bone marrow cells were isolated from the tibiae and femurs of untreated WT and AAT KO mice as recently described (8). Neutrophils were purified by using mouse neutrophil isolation kit (MACS Miltenyi Biotec, Bergisch Gladbach, Germany) according to manufacturer's instructions. Highly purified neutrophils and adherent rAM (2×10^5 cells per well) were infected with Spn at a multiplicity of infection (MOI) of 25 and incubated for 30 min in RPMI/10% FCS/1% glutamine with or without 50 µg/ml hAAT at 37°C and 5% CO_2. Following incubation, non-phagocytosed bacteria were removed by washing the cells three times, and remaining extracellular bacteria were killed by adding RPMI/10% FCS/1% glutamine +/- 50 µg/ml hAAT medium supplemented with gentamicin (20

µg/ml, Sigma Aldrich, St. Louis, USA) to the cells for approximately 10 minutes followed by washing of the cells and re-suspension in complete medium without antibiotics. To determine the phagocytosis and killing capacity of rAM and neutrophils of WT and AAT KO mice, cells were lysed at 30 min and 90 min post-infection and ten-fold serial dilutions were plated onto sheep blood agar plates, followed by incubation for 18 h at 37°C and 5% CO_2, after which CFU were enumerated.

Analysis of respiratory burst induction in bone marrow-derived neutrophils

Analysis of burst induction in bone marrow-derived neutrophils was done following recently published protocols (1, 6). Shortly, highly purified neutrophils (2 x 10^5) were resuspended in RPMI/10% and seeded per 96-well plate and allowed to rest for 30 minutes at 37°C/5% CO_2. After incubation, 2 mM luminol was added to each well and cells were again incubated for 1 h at 37°C/5% CO_2. Oxidative burst was then induced by adding Spn (MOI 5) or zymosan to the cells. Emitted luminescence was then recorded over time and is expressed as relative light units (RLU) using a Flx800 fluorescence/luminescence reader (BioTec Instruments, Bad Friedrichshall, Germany, KC4 software).

Lung histopathology

Mice were euthanized with an overdose of isoflurane followed by fixation of lung tissue via intratracheal instillation of 4 % formalin fixation medium (Roti®-Histofix, Carl Roth, Karlsruhe, Germany). Thereafter, lungs were removed en bloc and were immersed in fixation medium for at least 24 h, after which individual lung lobes were embedded in paraffin and sectioned at 2.5 µm with a microtome (Leica RM 2255). Lung tissue sections were then stained with hematoxylin/eosin and were examined with an Olympus BX53 microscope (Olympus, Tokyo, Japan) (1, 5).

Quantification of neutrophils in BAL and lung tissue

Identification and quantification of neutrophils recovered from BAL fluids and lung tissue of Spn-infected WT and AAT KO mice was done by flow cytometric analysis as described before (5, 7). Briefly, Spn-infected WT and AAT KO mice were subjected to BAL, followed by careful removal of lung lobes and digestion of lung tissue with collagenase A and DNAse I on day 3 post-infection. Leukocyte subsets purified from BAL fluids and lung homogenates were seeded onto 96-well plates (5×10^5 cells/well) and treated with Fc receptor blocking agent KIOVIG (Baxter AG, Vienna, Austria). Afterwards, cells were stained with fluorochrome-conjugated mAbs specific for CD45 (anti-CD45 PE-Cy7, clone 30-F11, BD Bioscience) and Ly6G V450 (anti-Ly6G, clone 1A8, BD Bioscience) followed by two washing steps. Subsequently, cells were subjected to FACS analysis of specific cell surface Ag expression using a BD LSR Fortessa flow cytometer (BD Biosciences, San Diego, CA).

References

1. Hahn I, Klaus A, Janze AK, Steinwede K, Ding N, Bohling J, et al. Cathepsin G and neutrophil elastase play critical and nonredundant roles in lung-protective immunity against Streptococcus pneumoniae in mice. *Infection and immunity.* 2011;79(12):4893-901.
2. Henken S, Bohling J, Martens-Lobenhoffer J, Paton JC, Ogunniyi AD, Briles DE, et al. Efficacy profiles of daptomycin for treatment of invasive and noninvasive pulmonary infections with Streptococcus pneumoniae. *Antimicrob Agents Chemother.* 2010;54(2):707-17.
3. Briles DE, Crain MJ, Gray BM, Forman C, and Yother J. Strong association between capsular type and virulence for mice among human isolates of Streptococcus pneumoniae. *Infection and immunity.* 1992;60(1):111-6.
4. Herbold W, Maus R, Hahn I, Ding N, Srivastava M, Christman JW, et al. Importance of CXC chemokine receptor 2 in alveolar neutrophil and exudate macrophage recruitment in response to pneumococcal lung infection. *Infection and immunity.* 2010;78(6):2620-30.
5. Steinwede K, Tempelhof O, Bolte K, Maus R, Bohling J, Ueberberg B, et al. Local delivery of GM-CSF protects mice from lethal pneumococcal pneumonia. *Journal of immunology.* 2011;187(10):5346-56.
6. Behler-Janbeck F, Takano T, Maus R, Stolper J, Jonigk D, Tort Tarrés M, et al. C-type Lectin Mincle Recognizes Glucosyl-diacylglycerol of Streptococcus pneumoniae and Plays a Protective Role in Pneumococcal Pneumonia. *PLoS Pathog.* 2016;12(12):e1006038.
7. Steinwede K, Henken S, Bohling J, Maus R, Ueberberg B, Brumshagen C, et al. TNF-related apoptosis-inducing ligand (TRAIL) exerts therapeutic efficacy

for the treatment of pneumococcal pneumonia in mice. *The Journal of experimental medicine.* 2012;209(11):1937-52.

8. Behler F, Maus R, Bohling J, Knippenberg S, Kirchhof G, Nagata M, et al. Macrophage-inducible C-type lectin Mincle-expressing dendritic cells contribute to control of splenic Mycobacterium bovis BCG infection in mice. *Infection and immunity.* 2015;83(1):184-96.

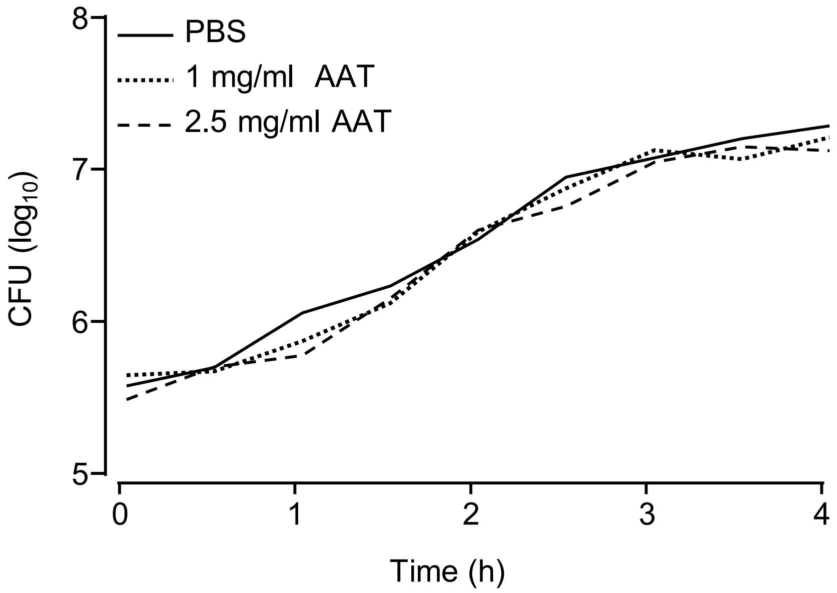

Figure S1: Growth kinetics of *S. pneumoniae* in the absence or presence of AAT. Spn was cultured in THB + 20 % FCS and bacterial growth was determined in the absence (PBS, solid line) or presence of AAT applied at concentrations of 1 mg/ml (dotted line) or 2.5 mg/ml culture medium (dashed line).

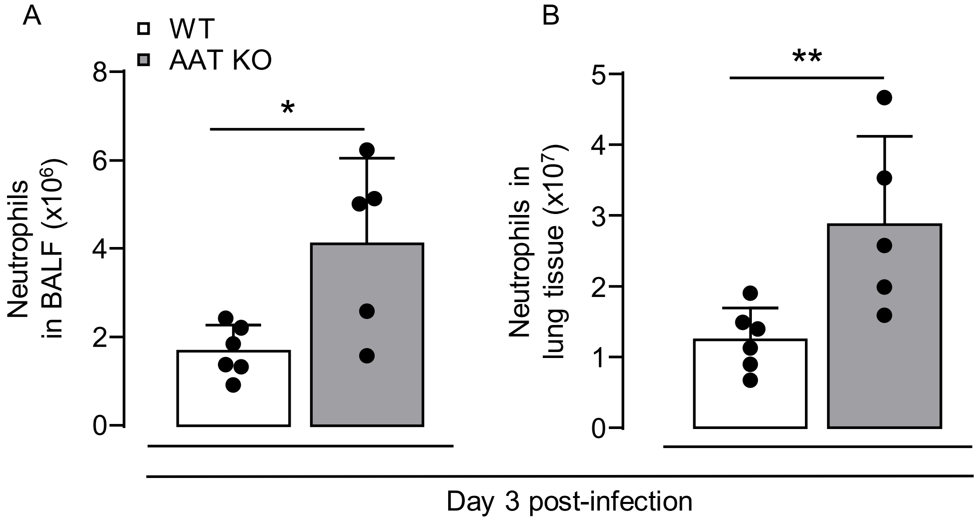

Figure S2: Bronchoalveolar neutrophil recruitment in *S. pneumoniae* infected WT versus AAT KO mice. WT mice (white bars) and AAT KO mice (grey bars) were infected orotracheally with Spn. (A,B) Numbers of alveolar neutrophils in BALF (A) and lung tissue (B) were determined by FACS analysis at 72 h post-infection. Values are presented as mean ± SD of n=5-6 mice per time-point and treatment group and are representative of two independently performed experiments. *p ≤ 0.05 and **p ≤ 0.01 compared to WT mice (Mann-Whitney U Test)

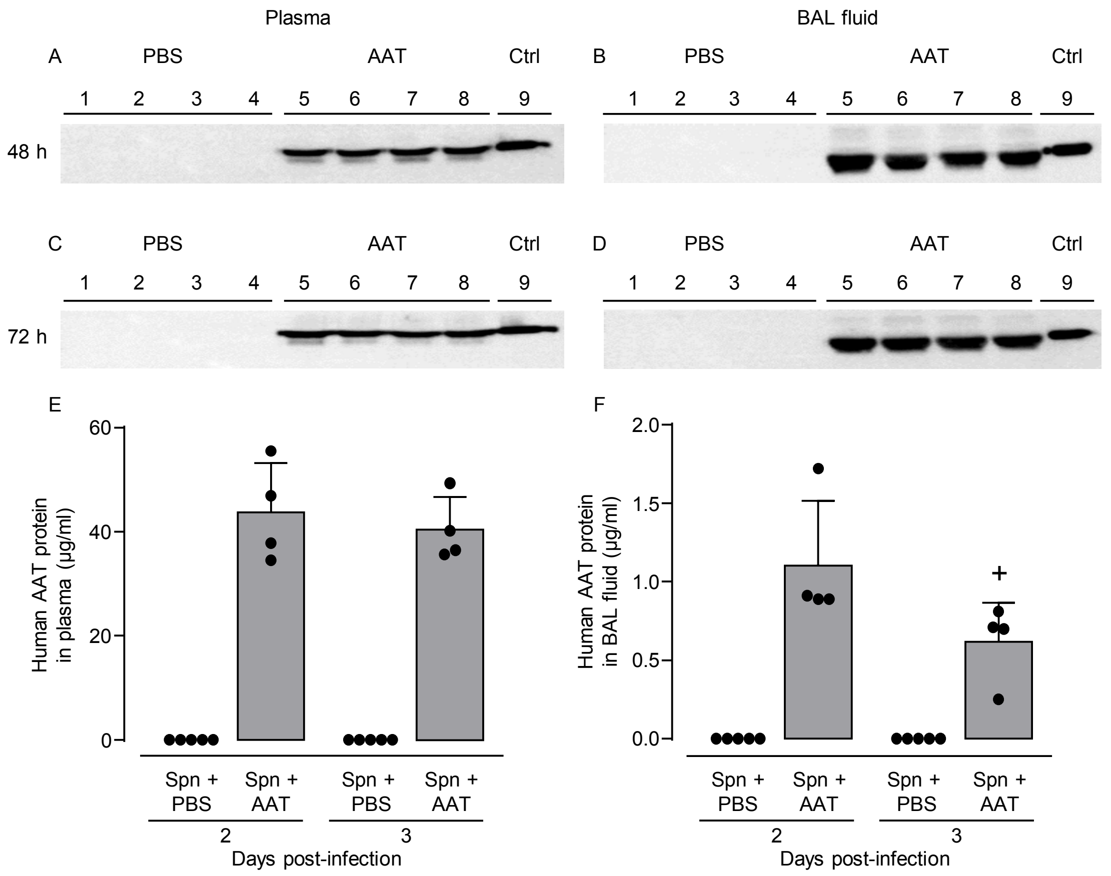

Figure S3: Exogenously applied human AAT is detectable in plasma and BAL fluids of AAT KO mice infected with *S. pneumoniae*. Plasma (A,C) and BALF samples (B,D) of Spn-infected AAT KO mice treated with vehicle (PBS, lanes 1 to 4) or Respreeza® (lanes 5 to 8) were subjected to Western Blot analysis of human AAT protein at 48 h and 72 h post-infection. Purified human AAT served as positive control (lane 9 in A-D). (E,F) Determination of hAAT protein levels (grey bars) in plasma (E) and BAL fluids (F) of mice by ELISA, as indicated. Data are shown as mean ± SD of n=4-5 mice per time-point and treatment group. +p ≤ 0.05 compared to day 2 (Mann-Whitney U test).
